# Correlations of Resource Bricolage and Exaptation With Low-Cost Breakthrough Innovations: Moderating Effect of Organizational Agility

**DOI:** 10.3389/fpsyg.2022.846629

**Published:** 2022-03-15

**Authors:** Chaoyong Tang, Yongzhi Shi, Ruilin Cai

**Affiliations:** ^1^School of Economics and Management, Agricultural University of Hebei, Baoding, China; ^2^Modern Educational Technology Center, Agricultural University of Hebei, Baoding, China; ^3^School of Business, Changshu Institute of Technology, Suzhou, China

**Keywords:** operational agility, marketing agility, low-cost breakthrough innovations, exaptation, resource bricolage

## Abstract

The mechanism influencing resource bricolage driving low-cost breakthrough innovations remains unclear. By introducing exaptation and organizational agility, this study creates a regulated mediation model, explores effects of resource bricolage on low-cost breakthrough innovations, and analyzes the moderating effect of organizational agility and mediation effect of exaptation. The results revealed that resource bricolage exerted a significant positive impact on low-cost breakthrough innovations, and exaptation played a mediation role between resource bricolage and low-cost breakthrough innovations. In addition, both marketing agility and operational agility positively regulated the correlation between resource bricolage and exaptation. Further research revealed that the mediation effect of exaptation was positively regulated by marketing agility and operational agility, respectively. Overall, this study enriches the discussion of the impact mechanism of breakthrough innovations by resource bricolage and provides valuable enlightenment for enterprises to implement innovation-driven development strategies in the context of economic transformation.

## Introduction

China, which is undergoing profound changes unseen in a century, faces both opportunities and challenges. China possesses a vast potential demand from the bottom of the pyramid (BOP) population in emerging markets, especially in the economic transition period when the phenomenon of technological homogeneity is serious (Zhao et al., 2019). Helping manufacturing enterprises to transform their innovation models has always been challenging. Conversely, the rapid reduction of imitation barriers and monopoly profits due to traditional innovation, as well as the rise of operating costs due to organizational complexity, and the reduction of organizational learning capabilities make it unsustainable. Hence, a vast gap exists between the potential demand of the BOP population and the supply of emerging markets. In this regard, how to develop high-quality and affordable products through low-cost breakthrough innovation with lower financial costs, shorter periods, and lower risks to establish their unique technology position, and stand out in the fierce competition is imperative (Zhu and Chen, 2014). Nevertheless, low-cost breakthrough innovations are often in the face of resource dilemmas, such as solidified resource concepts, backward management, poor configuration, and inadequate supply, which decrease the success rate of breakthrough innovations (Wang et al., 2019). Consequently, breaking out of the traditional resource management logic to promote low-cost breakthrough innovations is a crucial proposition that Chinese emerging market manufacturers urgently need to solve. As a new theory of resource management, resource bricolage can creatively use available resources, identify new attributes of resources through strategies like improvisation and restructuring, explore new uses of resources, and attain value creation of resources “from scratch” and “from less to more” through optimal allocation of resources and synergistic effects, thereby providing resource guarantee for breakthrough innovations (Liu and Wang, 2017; Xi et al., 2017; Huang, 2018). Nevertheless, the internal logic of resource bricolage to promote low-cost breakthrough innovations remains to be discussed. As the exaptation of the source of innovation, it provides the possibility to connect the relationship between the two. On the one hand, resource bricolage accentuates the deconstruction and reconstruction of available resources, including products and technology. By exploring and developing new functions of resources, resource bricolage reveals the use of resources as a method of discovering and creating opportunities (Yu et al., 2017), which, in turn, positively affects exaptation. On the other hand, exaptation can open up new industry space by transferring the existing products and technology or successful application in new fields, thereby promoting breakthrough innovations (Ren et al., 2019). Thus, this study constructs the influence mechanism of resource bricolage–exaptation–low-cost breakthrough innovations. Nevertheless, how will the above influence mechanism be affected by contingency? As a crucial dynamic capability, organizational agility covers the characteristics of identifying the market, quick response, and flexible adjustment, which can augment the foresight of the company’s strategy and the ability to make adjustments, changes, and rapid responses to future opportunities identification, and provide capacity guarantees for resource bricolage to promote breakthrough innovations through exaptation, thereby increasing the incidence and success rate of exaptation (Oosterhout et al., 2006). Hence, this study incorporates organizational agility into the theoretical model and further examines the boundaries of the role of resource bricolage–exaptation–low-cost breakthrough innovations.

The theoretical gap and novelty of this study are primarily reflected in the following aspects. First, the existing studies have investigated the impact of resource bricolage on new startups innovation and breakthrough innovation (Liu and Wang, 2017; Xi et al., 2017; Wang et al., 2019; Zhao et al., 2019). However, unlike the perspective of new startups, low-cost breakthrough innovation of manufacturing enterprises in emerging markets also needs the support of resource bricolage (Yu et al., 2017), but studies on the correlation between resource bricolage and low-cost breakthrough innovations are relatively deficient. This study supplements this limitation. Second, previous studies explored the mediating role between resource bricolage and breakthrough innovation from the viewpoints of entrepreneurial learning and dynamic capabilities (Liu and Wang, 2017; Huang, 2018), but few studies examined exaptation. This study focuses on the mediating mechanism of exaptation, thereby enriching the research on the formation path of low-cost breakthrough innovations. Finally, few studies examined the regulatory mechanism of organizational agility. With organizational agility as a moderating variable, this study reveals a contextual mechanism of resource bricolage influencing low-cost breakthrough innovations and enlarges the application fields of organizational agility.

## Theories

### Low-Cost Breakthrough Innovations

The existing problems in emerging markets are resource constraints, inadequate supply, strong demand, and weak purchasing power, making it challenging to effectively balance the imbalance between supply and demand in emerging markets by obeying the traditional breakthrough innovations model. Consequently, incorporating cost-effectiveness, inclusiveness, and redesign into breakthrough innovations to form low-cost breakthrough innovations paradigm has become a strategic choice for manufacturing companies to acquire competitive advantages. Zhu and Chen (2014) defined low-cost breakthrough innovations as an innovation by an organization that relies on new technology and knowledge or eliminates existing technological trajectories and knowledge, and uses low-cost design, technology, and organization to satisfy new markets and customers, and to further decrease low-cost *via* innovative methods. Zha et al. (2015) established that low-cost breakthrough innovations denote various feasible methods adopted by enterprises to decrease low-cost, to realize new products, services or technologies, pursue new knowledge, and attempt to break the original knowledge trajectory, which can introduce new products to open up new markets and provide new services for enterprises. This study claims that low-cost breakthrough innovations imply obeying the concepts of low-cost, redesign, tolerance, and openness, by pursuing new applications of knowledge and technology systems, to fulfill the needs of emerging markets BOP population to support the process of sustainable development of enterprises. Low-cost breakthrough innovations not only highlight the realization of major innovations and breakthroughs in product and technology based on low-cost strategy, thereby opening up new markets and gaining lasting competitiveness, but also focus on the integration of properties, such as low-cost, design thinking, openness, and inclusiveness, based on breakthrough innovations to provide BOP population with better-quality products and services. Previous studies have mostly investigated the formation path of low-cost breakthrough innovations from the standpoints of knowledge governance (Zhu and Chen, 2014), failure learning, and intellectual capital (Zha et al., 2015), but rarely involve issues on how to crack the resource constraints of low-cost breakthrough innovations in emerging markets.

### Resource Bricolage

Bricolage is defined as the process of coalescing resources at hand and acting immediately to address new problems and discover new opportunities (Baker and Nelson, 2005). The core of bricolage is the re-deconstruction and integration of existing elements to create new rules and methods (Lévi-Strauss, 1997), which possesses fundamental characteristics such as available resources, resource improvisation, rapid response, action preference, and resource reconstruction. Based on the results of bricolage of human culture and mind and its application practices, this study argues that the objects of bricolage are all resources of enterprises, and bricolage is an “improvisation” to satisfy market opportunities and a crucial guiding ideology. Besides, it is a decision-making tool that aligns with the enterprise development strategies. The essence of bricolage is innovation, openness, sharing, and creation, and the bricolage are elementary elements like resource awareness, satisfactory decision-making, and resource reconstruction. Of note, bricolage aims to gain enterprise competitive advantages and sustainable development. According to the existing research, with the integration of bricolage theory into the innovation process, bricolage has become a crucial path for value creation, which is also a critical way to address the constraints of technology, personnel, and material resources and help new enterprises achieve technological innovation (Wang et al., 2019; Zhao et al., 2019). Furthermore, some studies highlighted that resource bricolage exerts a positive impact on breakthrough innovations of startups (Liu and Wang, 2017; Xi et al., 2017; Huang, 2018). However, prior studies have seldom discussed the logic through which resource bricolage affects the low-cost breakthrough mechanism and path.

### Exaptation

As a source of innovation, exaptation originated initially in evolutionary biology, denoting the evolution of certain entities for other uses, and then replacing their original uses afterward (Gould and Vrba, 1982). Mokyr (2011) transplanted exaptation to management and interpreted the phenomenon that “a certain technology is accidentally used in another field and becomes successful” as exaptation. The concept of exaptation comprises two main points: function transfer and accidental occurrence. Of these, function transfer covers the transfer of product functions and technologies, primarily implying products or technologies that have attained new functions and resolved new problems in a new environment. Accidental occurrence stresses that exaptation is an unpredicted result or understood as a reverse innovation mechanism that looks for questions with answers at hand, which has some degree of contingency and risk. Nevertheless, the likelihood of exaptation can be increased by adopting an innovative management model, fostering an innovative culture, augmenting industry–university–research cooperation, and enhancing learning ability. Previous studies illustrated that institutional interactions (Garud et al., 2018), diverse knowledge reserves, cultural innovations, and organizational structures of internal and external linkage (Andriani et al., 2017) exerted a positive predictive impact on exaptation. Moreover, the new functions of exaptation stemmed from the rearrangement and recombination of the existing components into a new and complex form (Gregory, 2008), revealing the key role of bricolage decomposition and reconstruction strategy in the formation of exaptation. As an analytical tool with strategic value, exaptation accentuates the development and transfer of new functions, or seeks to apply the existing products or technologies in new fields to produce unanticipated creative results and increment of value, thereby creating a new technological trajectory, market, or industry (Andriani and Carignani, 2014; Ren et al., 2019), which has positive value for breakthrough innovations. Hence, exaptation might play a crucial role in the process of resource bricolage-driven breakthrough innovations. However, the existing research lacks attention to this, and how exaptation connects the relationship between the two warrants further exploration.

### Organizational Agility

As a high-level dynamic capability, organizational agility denotes the organization’s ability to sensitively perceive swift changes in the outside world and respond to changes timely, formulate new strategies flexibly, quickly, and effectively, and integrate and allocate its own resources (Wang and Li, 2018). The organizational agility comprises marketing and operational agility (Lu and Ramamurthy, 2011), among which marketing agility reflects an organization’s ability to satisfy customer needs through continuous monitoring and rapid enhancement of products or services, and respond quickly to changes or take advantage of changes; whereas, operational agility reflects the ability to alter internal business processes within the enterprise to quickly respond to market changes. Organizational agility can not only help enterprises perceive and respond to consumer demand preferences, competitor strategies, industry developments, and changes in the macro-environment, precisely capture opportunities in environmental changes or new application prospects, and select a new trigger scenario for the realization of exaptation, but also help enterprises redesign products, technologies, and business processes (Teece et al., 2016), develop the potential usability of the existing products and technologies to perform a new function, and then promote breakthrough innovations through exaptation.

## Hypotheses

### Resource Bricolage and Low-Cost Breakthrough Innovations

Previous studies established a positive correlation between innovations and resource bricolage with its elementary strategy of “available resources, immediate action, and resource reconstruction” (Garud and Karnøe, 2003; Baker and Nelson, 2005; Senyard et al., 2014). Meanwhile, enterprises with severe resource limitations can not only help promote innovation but also breakthrough innovations through strategies like identifying the heterogeneous characteristics of resources and realizing the creative restructuring of resources (Liu and Wang, 2017). Garud and Karnøe (2003) explored Danish and American wind turbine cases and reported that compared with the traditional plan followed by the United States, Denmark adopted a bricolage strategy of integrated development of available resources (including idle resources) to advance wind turbine technology and, thus, made breakthrough progress, revealing that the application of seemingly impractical resources or universal resources through creative bricolage strategies could generate unexpected value, thereby attaining high-level innovation in a low-cost manner. Thus, how to enhance low-cost breakthrough innovations? Resource bricolage is a feasible starting perspective of this question. On the one hand, the research on the internal attributes of resources at hand has furthered comprehensively, and new values of resource attributes are identified and refined. Meanwhile, resource bricolage is committed to reorganizing functions and rearranging structures to attain a systematic reconfiguration of resources, resulting in previously unpredicted breakthrough technology (Garud and Karnøe, 2003). On the other hand, resource bricolage has the attribute of action preference (Baker and Nelson, 2005), following the “satisfaction principle,” which underlines quick response to immediate action, without waiting for multiple investigations and resource reserve processes, thereby making full use of the limited resources at hand to accomplish innovation (Baker and Nelson, 2005). Besides, its other characteristics are low time cost, low financial cost, and inclusivity. As mentioned elsewhere (Xi et al., 2017), it is essential to integrate and utilize available resources, explore new value attributes of resources, restructure resources, and constantly excavate and create new knowledge and new technologies in the process of bricolage-driven innovation; the former has innovative cost effectiveness, while the latter has innovative breakthroughs. Overall, resource bricolage can realize the structural re-decomposition and restructuring of available resources, which focuses more on the exploration and utilization of available resources and provides creative ideas for the realization of breakthrough innovations. Hence, the following hypothesis is proposed:

**H1:** Resource bricolage exerts a positive impact on low-cost breakthrough innovations.

### Mediation Effect of Exaptation

The exaptation aims to transfer functions and discover new application fields of products or technologies (Garud et al., 2018). In technological innovation, resource bricolage can realize the exploration or rearrangement of the existing technologies into a new and more complex form, thereby generating new functions, enabling enterprises to attain unexpected and innovative results (Banerjee and Campbell, 2009); it reveals that resource bricolage is a crucial way to activate and promote exaptation. First, the accretion of diverse knowledge helps to create exaptation (Garud et al., 2018). Enterprises can foresee unpredicted new environments and explore future opportunities by accruing knowledge. On the one hand, resource bricolage is an organizational learning process, which needs breaking conventions and examining and trying different methods, as well as new interpretations in new contexts, and endowing resources (technology or product) new meaning, value, and functions. On the other hand, resource bricolage can assimilate knowledge in different fields, excavate and reveal unused technical features and functions, and create opportunities to be applied to new application fields (Andriani and Carignani, 2014). Moreover, expanding knowledge in different fields would help R&D personnel have a more comprehensive understanding of products and technology, increasing the likelihood of successful exaptation. Second, the technical complexity of a product is a crucial condition for promoting expansion activities. Communication and interaction are the fundamental elements of bricolage (Lévi-Strauss, 1997). In the bricolage process of experimentation and exploration, enterprises, along with exchanges and discussions among different knowledge and skilled personnel, can promote the flow and sharing of knowledge and increase the possibility of technical decomposition and combination, thereby enhancing the prospect of exaptation (Andriani et al., 2017). Third, institutional interaction is a crucial prerequisite for the formation of exaptation (Garud et al., 2018). At the organizational level, resource bricolage is manifested as the interaction between enterprises and various institutions, including customers, intermediaries, suppliers, competitors, and mediating service organizations, exhibiting cognition diversity, the connections of organizational resources and capabilities. Through the innovation, integration, and reconstruction of the related knowledge, resource bricolage assists in realizing the new functional applications of products and technologies. Accordingly, the following hypothesis is proposed:

**H2:** Resource bricolage exerts a positive impact on exaptation.

The essence of exaptation is the successful application of the existing technologies under new conditions or in new fields, or the combination of knowledge in the existing technical fields to enhance absorptive capacity, create novel and valuable new products, and open up a new market or technological trajectory (Ren et al., 2019). Andriani et al. (2017) reported that as the market distance from the original product increased, the likelihood of examining the unknown increased, while the likelihood of competition declined. Moreover, the new functions due to exaptation depend on new market scenarios or trigger mechanisms, allowing meeting previously unrealistic conditions and even discovering new demands to help companies become industry pioneers. In addition, Andriani et al. (2017) proposed that each drug in the pharmaceutical industry has 2.2 new types, one of which also has a strong breakthrough. Precisely, each product contains potential options that can be transformed into new markets, revealing that the combination of existing products and appropriate scenarios might induce function transfer, resulting in breakthrough innovations. Raytheon company found that the magnetron of the radar component has a new function of melting candies, which was then applied for a patent in 1947, and launched the world’s first microwave oven (Osepchuk, 1984), thereby opening up a new market. This case reveals the mechanism of action of exaptation driving low-cost breakthrough innovations. Exaptation is primarily based on the examination of the existing technology or product, by enhancing knowledge screening in the process of innovation and the efficacy of combination and utilization, which scarifies and excavates its potential functions, or redesigns the internal function module within products to realize function transfer, resulting in the development of a new market and driving low-cost breakthrough innovations. Conversely, compared with developing a new product, exaptation is established in the familiar technology field, which not only possesses more economical efficiencies through the extraction of the potential value of the existing product or technology but also increases the resources interest rate, creates the new knowledge combination and the question solving, and promotes advanced innovation in a low-cost manner through the redevelopment and redesign of the available resources. Accordingly, the following hypothesis is proposed:

**H3:** Exaptation exerts a positive impact on low-cost breakthrough innovations.

Combining H2 and H3, this study argues that exaptation will mediate the correlation between resource bricolage and low-cost breakthrough innovations. Hence, the following hypothesis is proposed:

**H4:** Exaptation has a mediation effect between resource bricolage and low-cost breakthrough innovations.

### The Regulating Effect of Organizational Agility

As a distinct dynamic ability of enterprises, organizational agility aids in suppressing inertial thinking, as well as break through the limitations of organizational conventions and reflect on strategic goals and its operational processes. Moreover, it helps to innovate organizational structure and operational capabilities, and actively promote innovative practices (Sanchez, 2010; Lee et al., 2015). Cai et al. (2019) illustrated that organizational agility can sense market changes, readily transmit the early warning information to enterprises, endorse the integration of resources and structures and the innovation of product and business process, to play a regulatory role in bricolage driving exaptation. This study demonstrates the adjustment mechanism between resource bricolage and exaptation from the two dimensions of marketing agility and operational agility (Lu and Ramamurthy, 2011). Under the conditions of high-marketing agility, enterprises good at resource bricolage can not only quickly capture market opportunities, develop new attributes and new functions of product and technology but are also more inclined to seek new application field or investigate new application value for the existing product or technology, thereby promoting the exaptation process. In contrast, under the condition of low-marketing agility, enterprises are more likely to disregard the demand changes, industry, and macro-environmental dynamics, resulting in an inability to make full use of the potential advantages of the existing technology or product, and challenges to provide driving conditions for exaptation, and are thus unable to use existing solutions to explore opportunities for future benefits. Second, when enterprises with high-level operational agility conduct resource bricolage, they can quickly alter the organizational structure and operating processes based on market changes, update organizational routines, innovate management models, eliminate institutionalized mindset (Ganzaroli et al., 2014), and form an interdependent, coordinated, and open management mechanism. In addition, by rearranging the existing product or technology into a new form, they can examine new functions of the product or technology, which thus promotes the exaptation process. Conversely, enterprises with low-operational agility often rely on experiences like traditional mindset and organizational inertia that stick to the past, weakening the organizational structure renewal, business process adjustment, and innovation culture construction process, hampering the creation of ecological network chains, such as enterprises, users, and communities, as well as the effective communication between resource capabilities of enterprises and external networks, thereby decreasing the likelihood of new product and technology development (Andriani et al., 2017). Accordingly, the following hypotheses are proposed:

**H5a:** Marketing agility positively regulates the correlation between resource bricolage and exaptation.
**H5b:** Operational agility positively regulates the correlation between resource bricolage and exaptation.

Based on these hypotheses, organizational agility (marketing agility and operational agility) positively regulates the correlation between resource bricolage and exaptation (H5a/H5b), and exaptation exerts a mediation effect between resource bricolage and low-cost breakthrough innovations (H4). This study deduces that organizational agility (marketing agility and operational agility) would regulate the mediation effect of exaptation between resource bricolage and low-cost breakthrough innovations, creating a regulated mediation model. Specifically, the higher the level of organizational agility (marketing agility and operational agility), the greater the positive impact of resource bricolage on low-cost breakthrough innovations by promoting exaptation. Hence, the following hypotheses are proposed:

**H6a:** Marketing agility positively regulates the mediation effect of exaptation in the correlation between resource bricolage and low-cost breakthrough innovations.
**H6b:** Operational agility positively regulates the mediation effect of exaptation in the correlation between resource bricolage and low-cost breakthrough innovations.

**FIGURE 1:**
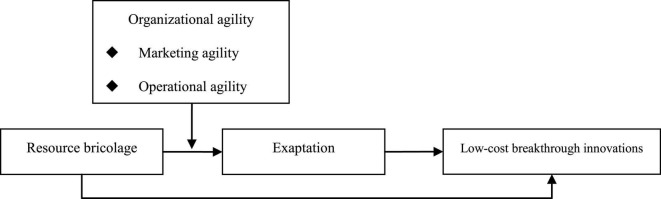
Theoretical model.

Figure 1 shows the theoretical model of this study, specifically, resource bricolage exerts a positive impact on low-cost breakthrough innovations, resource bricolage exerts a positive impact on exaptation, exaptation exerts a positive impact on low-cost breakthrough innovations, exaptation has a mediation effect between resource bricolage and low-cost breakthrough innovations, marketing agility positively regulates the correlation between resource bricolage and exaptation, operational agility positively regulates the correlation between resource bricolage and exaptation, marketing agility positively regulates the mediation effect of exaptation in the correlation between resource bricolage and low-cost breakthrough innovations, operational agility positively regulates the mediation effect of exaptation in the correlation between resource bricolage and low-cost breakthrough innovations.

## Research Design

According to Chen et al. (2008), it is necessary to select an appropriate research method according to the nature of the problem when conducting research design. The current commonly used research methods comprise an experimental method, questionnaire survey, secondary data, and qualitative research, which have no advantages or disadvantages. Thus, the choice of method should be based on the researcher’s understanding of the research question. This study primarily examines “the correlation among resource bricolage, exaptation, and low-cost breakthrough innovation.” The research hypothesis is at the organizational level. As the independent variables are difficult to control or manipulate, the experimental method is not an effective method for this research; owing to the lack of access to secondary data, this method is not applicable. Qualitative research requires researchers to have extensive contact with the research object, the sample size should be small, and it is hard to repeat verification; hence, this method is unsuitable for this study. The questionnaire survey method is fast, effective, cost-effective, less interfering to subjects, easy to obtain support, and, at the same time, large-scale samples can ensure sufficient variation of independent variables; thus, questionnaire survey is the most suitable method for this study.

### Sample Data Collection

This study primarily selected the emerging markets manufacturing enterprises in Shanxi province, Jiangsu province, and other regions of China as the research samples and emphasized their academic and anonymous characteristics on the front page of the questionnaire. The research objects included managers and core technical personnel owing to their deeper understanding of issues such as enterprise technology innovation strategy, marketing strategy, resource bricolage strategy, and operation management, and better ability to understand the practice of bricolage driving breakthrough innovations. First, a small-scale enterprise sample was selected to test the accuracy, logic, consistency, and refinement of the questionnaire content, based on which the validity of the questionnaire was improved. Second, we used two research methods: onsite and MBA. For the former, it was primarily done by contacting the tested enterprises, negotiating time slot, location, and related research personnel, and adopting onsite or entrusted methods. For the latter, the questionnaire survey was largely arranged during class breaks, which was completed by onsite recovering. A total of 507 questionnaires were distributed, of which 420 were issued by onsite research and 348 were recovered, and 87 were issued by MBA and 73 were recovered. A total of 58 invalid questionnaires, such as incomplete answers and logical errors, were eliminated, and 363 valid questionnaires were finally obtained. The effective recovery rate was 86.22%. The sample profile was as follows: regarding enterprise age, 5–10 years accounted for 5.79%, 11–15 years for 18.18%, 16–20 for 27.82%, 21–30 for 35.26%, and >30 years for 12.95%. Regarding enterprise scale, ≤100 people accounted for 1.10%, 100–499 accounted for 13.22%, 500–1,000 accounted for 26.72%, 1,001–2,000 for 44.08%, and ≥2,000 for 14.88%. Regarding industry, food processing accounted for 3.03%, textiles and apparel accounted for 11.02%, furniture manufacturing for 19.56%, pharmaceutical manufacturing for 53.99%, and others for 12.40%. Moreover, to avoid the problem of homology deviation, the Harman method was used for inspection, and the results revealed that the first factor explained 32.226% of the variation under the unrotated condition, which is <50%, indicating no significant common deviation in the data.

### Variable Measurement

Based on the literature review, appropriate corrections were made to determine the measurement items. The questionnaire was designed with a 5-point Likert scale, in which 1 = “strongly disagree” and 5 = “strongly agree.” Table 1 presents the specific measurement items of each main variable.

**TABLE 1:** Factor analysis and the results of reliability and validity.

Variable	Measurement item	Factor load	Cronbach α	AVE
Resource bricolage	Enterprises combine resources by challenging traditional business practices	0.832	0.721	0.648
	Enterprises combine resources by obtaining value from resources that have never been fully utilized	0.827		
	Enterprises allocate resources in a way that they can obtain innovative solutions	0.753		
Exaptation	A technology of the enterprises has been successfully applied in another field	0.885	0.833	0.750
	A product of the enterprises has gained new functions in the new environment	0.883		
	Enterprises have new functions through the combination of existing product functions	0.830		
Marketing agility	When the external market changes, enterprises quickly make and implement appropriate decisions.	0.813	0.677	0.608
	Enterprises are constantly looking for methods to restructure their organizational structure to better serve the market.	0.796		
	Enterprises consider market-related dynamic changes as opportunities for development	0.727		
Operational agility	Enterprises quickly adjust production/service levels to respond to fluctuations in market demand	0.822	0.679	0.610
	Whenever the supplier’s supply is interrupted, enterprises can quickly make a reasonable decision to solve the problem	0.799		
	Enterprises are able to satisfy customer requirements for quick response	0.719		
Low-cost breakthrough innovations	Enterprises make full use of available resources to develop new products (services)	0.757	0.755	0.507
	Enterprises develop new technologies and products with low-cost design	0.749		
	Enterprises have opened up new markets with new products/services	0.740		
	Enterprises fundamentally change their leading products or services in a low-cost manner	0.689		
	Enterprises are famous for their low-cost breakthrough innovations products	0.615		

Resource bricolage uses available resources to act immediately and refactor and innovate. As reported elsewhere (Salunke et al., 2013), resource bricolage comprises three items. Exaptation highlights the function transfer of the enterprise’s existing product and technology, or the application of the existing technology in new fields. As reported elsewhere (Mastrogiorgio and Gilsing, 2016), exaptation comprises three items. Organizational agility reflects the agility of the enterprise’s perception and response in terms of operational adjustment and market utilization. As reported previously (Lu and Ramamurthy, 2011), organizational agility comprises two dimensions: marketing agility (three items) and operational agility (three items). Low-cost breakthrough innovations develop and promote new products based on maximizing the use of enterprise resources to fulfill users’ basic demands. As reported elsewhere (Zhu and Chen, 2014; Zha et al., 2015), low-cost breakthrough innovations comprise five items. Furthermore, this study drew on the practices of previous studies (Xi et al., 2017) and incorporated enterprise age, industry distribution, and enterprise size as control variables into the research model.

## Data Analysis and Results

### Reliability and Validity Test and Correlation Analysis

First, regarding reliability, based on the internal consistency of Cronbach α value test scale, the results suggested that the Cronbach α values of resource bricolage, exaptation, marketing agility, operational agility, and low-cost breakthrough innovations were 0.721, 0.833, 0.677, 0.679, and 0.755, respectively, which were all greater than or close to 0.7, suggesting that the scale has good internal consistency and high reliability. Second, regarding the validity, parts of the scales denote or use relevant research results published in high-quality journals at home and abroad. Meanwhile, a “double-blind” translation was conducted in the designing process of the scale to ensure the scale’s content validity. Meanwhile, using the average variance extraction (AVE) as a convergence validity test tool, the AVE values of resource bricolage, exaptation, marketing agility, operational agility, and low-cost breakthrough innovations were 0.648, 0.750, 0.608, 0.610, and 0.507, respectively, which are all >50%, suggesting that the main variables had good convergence validity. In addition, the square root value of the AVE value of each variable was higher than the corresponding correlation coefficient value (Table 2 shows the diagonal bold data), suggesting that the scale had good discrimination validity. Moreover, regarding the correlation of variables, resource bricolage positively correlated with low-cost breakthrough innovations based on the results of the correlation test (Table 2), and the rationality of H1 can be preliminarily judged. A positive correlation was found among resource bricolage, exaptation, and low-cost breakthrough innovations. Thus, it can be preliminarily judged that exaptation could have a mediation effect between resource bricolage and low-cost breakthrough innovations, suggesting the rationality of assumptions H2, H3, and H4. Furthermore, a positive correlation was found among organizational agility (marketing and operational agility), exaptation, and low-cost breakthrough innovations, suggesting that H5a, H5b, H6a, and H6b have certain feasibility.

**TABLE 2:** Mean, standard deviation, and correlation coefficient.

Variable	1	2	3	4	5
Resource bricolage	**0.805**				
Exaptation	0.363**	**0.866**			
Marketing agility	0.431**	0.283**	**0.780**		
Operational agility	0.524**	0.253**	0.553**	**0.781**	
Low-cost breakthrough innovations	0.458**	0.358**	0.436**	0.460**	**0.712**
Mean	4.322	3.615	4.071	4.176	4.145
Standard deviation	0.510	1.046	0.577	0.553	0.555

**Significant at the 0.01 level; *significant at the 0.05 level (two-tailed test).

### Hypotheses Testing

#### Main Effect Test

Table 3 shows the test results. According to Table 3, Model 2, resource bricolage exerts a significant positive impact on low-cost breakthrough innovations (β = 0.461, *P* < 0.001), assuming H1 holds. It demonstrates that resource bricolage is a crucial basis for driving low-cost breakthrough innovation. Through resource bricolage, the existing resources can be integrated, resource value creation is attained through strategies and reconstruction measures, and new resource generation and creation logic are provided for breakthrough innovation.

**TABLE 3:** Regression analysis results.

Variable	Exaptation	Low-cost breakthrough innovations			Exaptation			
	Model 1	Model 2	Model 3	Model 4	Model 5	Model 6	Model 7	Model 8
Enterprise age	−0.076	0.011	0.043	0.028	−0.077	−0.078	−0.078	−0.083
Enterprise scale	−0.002	0.057	0.041	0.057	−0.002	−0.006	−0.001	−0.003
Industry distribution	−0.024	−0.075	−0.062	−0.070	−0.023	−0.022	−0.0326	−0.022
Resource bricolage	0.363***	0.461***		0.380***	0.296***	0.328***	0.317***	0.353***
Exaptation			0.362***	0.223***				
Marketing agility					0.156**	0.182**		
Resource bricolage × marketing agility						0.121*		
Operational agility							0.089	0.134*
Resource bricolage × operational agility								0.157**
Δ*R*^2^	0.138	0.217	0.136	0.260	0.158	0.170	0.144	0.163
*F*	14.336***	24.871***	14.044***	25.134***	13.384***	12.162***	11.992***	11.585***

***Significant at the 0.001 level; **significant at the 0.01 level; *significant at the 0.05 level.

#### Mediating Effect Test

Table 3, Model 1 demonstrates that resource bricolage contributes to exaptation (β = 0.363, *P* < 0.001), assuming H2 holds. It demonstrates that bricolage is a crucial predictor of exaptation. Diverse knowledge can be accrued, and an open network that brings internal and external links creates conditions for adaptation *via* bricolage. Model 3 tests the correlation between exaptation and low-cost breakthrough innovations, suggesting is a positive correlation between the two (β = 0.362, *P* < 0.001), assuming H3 holds. It demonstrates that the innovation generated by adaptation has the characteristics of low cost and breakthrough. According to Model 4, when the independent variable and the mediating variable enter the regression model simultaneously, the mediating variable exaptation exerts a positive and significant impact on low-cost breakthrough innovations (β = 0.223, *P* < 0.001), whereas the impact of resource bricolage on low-cost breakthrough innovations is significant and smaller (β = 0.380, *P* < 0.001), suggesting a partial mediating effect, assuming hypothesis H4 holds. It shows that expansion adaptation is an important path for resource bricolage to drive low-cost breakthrough innovation. It is revealed that adaptation is a key path for resource bricolage to drive low-cost breakthrough innovation.

To compensate for the possible instability of the research conclusions due to a single test method, we adopted the bias-corrected non-parametric percentage Bootstrap test to validate the mediation effect of exaptation, which repeated the sampling 2,000 times and constructed a 95% unbiased calibration confidence interval (CI). The mediating effect of exaptation was 0.080, and 95% CI was [0.041, 0.129], excluding 0, indicating that exaptation exerted a significant mediation effect. Besides, the direct effect of resource bricolage on low-cost breakthrough innovations was 0.373, and 95% CI was [0.278, 0.468], excluding 0, further suggesting that exaptation has a mediation effect in the process of low-cost breakthrough innovations driven by resource bricolage, assuming that H4 is further verified.

#### Moderating Effect Test

**FIGURE 2:**
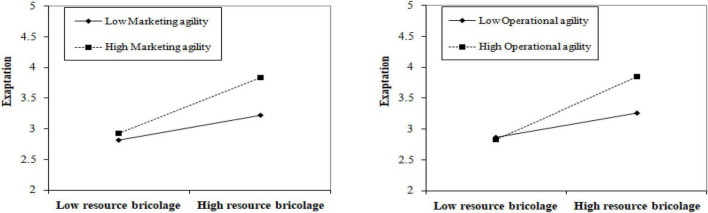
The moderating effect of organizational agility on the correlation between resource bricolage and exaptation.

First, according to Table 3, Models 5 and 6, the product term of resource bricolage and marketing agility was significant and positive (β = 0.121, *P* < 0.05), suggesting that marketing agility positively regulates the correlation between resource bricolage and exaptation, assuming that H5a holds. Precisely, the higher the market agility, the stronger the ability of enterprises to capture and utilize external opportunities, which is more helpful to realize the coupling of internal knowledge and external opportunities with the function of resource bricolage, and to augment the process of adaptation. According to Table 3, Models 7 and 8, the product term of resource bricolage and operational agility is significant and positive (β = 0.157, *P* < 0.01), suggesting that operational agility positively regulates the correlation between resource bricolage and exaptation, assuming that H5b holds. Thus, the higher the operational agility is, the more conducive it is to modify business processes and innovate practices through resource bricolage, realize product recombination or generate new functions, and accelerate the process of adaptation. Meanwhile, this study drew the moderating effect diagram of organizational agility (Figure 2); the higher the level of organizational agility, the stronger the impact of resource bricolage on the adaptation, and the slope of the straight line of high-level organizational agility is significantly higher than that of the low-level organizational agility. Second, the Bootstrap method was used to further test the moderating effect of organizational agility. Accordingly, the interaction coefficient between resource bricolage and marketing agility was significant (β = 0.329, *P* < 0.05; 95% CI [0.084, 0.690]), excluding 0, where the indirect effect was significant when marketing agility was low (*r* = 0.405; BootLCI [0.171, 0.638]); while at high-marketing agility, the indirect effect was significant (*r* = 0.816; BootLCI [0.514, 1.117]), assuming that H5a is verified further. Likewise, the interaction coefficient between resource bricolage and operational agility was significant (β = 0.384, *P* < 0.01; 95% CI [0.118, 0.651]), excluding 0, where the indirect effect was significant when operational agility was low (*r* = 0.427; BootLCI [0.189, 0.665]); while at high-operational agility, the indirect effect was significant (*r* = 0.889; BootLCI [0.595, 1.182]), assuming that H5b is verified further.

#### Test of Regulated Mediating Effect

The mediating effect of exaptation between resource bricolage and low-cost breakthrough innovations was analyzed when the level of organizational agility was different, where the Bootstrap method was used for 2,000 repeated sampling of samples and the CI was 95%. Table 4 presents the test results. The results revealed that at the low-marketing agility level, the mediating effect of exaptation was established with 95% CI [0.021, 0.091], while at the high-marketing agility level, the mediating effect of exaptation was established with 95% CI [0.050, 0.164]. Besides, the test results of the regulated mediating effect showed that its index value was 0.039 (95% CI [0.012, 0.081]), excluding 0, suggesting that the regulated mediation model was established, assuming that H6a is verified. Thus, the higher the market agility, the stronger the mediating effect of adaptation. Similarly, at the low-operational agility level, the mediating effect of exaptation was established (95% CI [0.024, 0.095]), while at the high-operational agility level, the mediating effect of exaptation was established (95% CI [0.051, 0.176]). The test results of the regulated mediating effect demonstrated that its index value was 0.045 (95% CI [0.012, 0.099]), excluding 0, suggesting that the regulated mediation model was established, assuming that H6b is verified. The higher the operational agility, the stronger the mediating effect of adaptation.

**TABLE 4:** Test results of the regulated mediating effect.

Mediation variable	Moderator	Indirect effects under different conditions				Regulated mediating effect			
		Effect	SD	Lower limit set	Upper limit	Index	SD	Lower limit set	Upper limit
Exaptation	Low-marketing agility	0.048	0.017	0.021	0.091	0.039	0.018	0.012	0.081
	High-marketing agility	0.096	0.028	0.050	0.164				
Exaptation	Low-operational agility	0.050	0.017	0.024	0.095	0.045	0.022	0.012	0.099
	High-operational agility	0.104	0.032	0.051	0.176				

## Discussion and Conclusion

### Conclusion

Under the background of economic transformation, low-cost breakthrough innovations are essential to respond to the demand of emerging markets BOP population; however, how to break through resource restrictions to attain low-cost breakthrough innovations is a strategic proposition fronted by emerging markets manufacturers. This study focused on the practices of low-cost breakthrough innovations in the Chinese context, investigating the impact mechanism of resource bricolage on low-cost breakthrough innovations and used exaptation as the mediating variable and organizational agility as the adjustment variable to build a regulated mediation model. The findings revealed that resource bricolage helps enhance low-cost breakthrough innovations, and exaptation mediates the correlation between resource bricolage and low-cost breakthrough innovations. Besides, organizational agility (marketing agility/operational agility) positively regulates the correlation between resource bricolage and exaptation, which also regulates the mediation effect of exaptation between resource bricolage and low-cost breakthrough innovations. The theoretical contributions of this study are as follows.

### Theoretical Contributions

(1) The research context of the impact of bricolage on breakthrough innovations is augmented. Previous studies on the impact of bricolage on breakthrough innovations focused on the aspects of bricolage on dual innovations and its synergy (Liu and Wang, 2017; Xi et al., 2017; Huang, 2018), with limited studies focusing on how bricolage affects low-cost breakthrough innovations. The discovery that bricolage promotes low-cost breakthrough innovations established in this study not only echoes and extends the research conclusions about the correlation between bricolage and breakthrough innovations (Gould and Vrba, 1982; Xi et al., 2017; Huang, 2018) but also reveals that emerging markets manufacturing enterprises urgently need a feasible path for low-cost breakthrough innovations, which relies on resources bricolage to alter the concept of resource management and breakthrough resource constraints. In addition, unlike previous studies that applied resource bricolage to the innovative practices of entrepreneurial enterprises (Liu and Wang, 2017; Zuo and Zhou, 2017; Huang, 2018), this study associates the characteristics of emerging markets, bricolage mindset, and breakthrough innovations to draw a research conclusion on bricolage to promote low-cost breakthrough innovations, illustrating the value of bricolage in promoting low-cost breakthrough innovations and its feasibility of application in innovative practices as valued by emerging markets manufacturing enterprises.
(2) The internal mechanism of the impact of resource bricolage on low-cost breakthrough innovations is revealed. This study provides a new theoretical perspective for opening the “black box” of resource bricolage affecting low-cost breakthrough innovations, revealing that exaptation can play a vital role in the correlation between resource bricolage and low-cost breakthrough innovations, interpreting the intrinsic mechanism of bricolage driving low-cost from a new outlook. Under the background where breakthrough innovations path supported by core competence theory is being challenged (Liu and Wang, 2017; Xi et al., 2017; Huang, 2018; Zhao et al., 2019), as a reverse innovation and strategic cognitive tool (Ren et al., 2019), exaptation highlights the use of the existing products or technologies to investigate new functions, new applications, or opening up new fields, which open up a new technological trajectory for enterprises, thereby cultivating breakthrough innovations (Andriani et al., 2017). Meanwhile, exaptation is also inseparable from the integration of knowledge and the promotion of reconstruction and innovation in various fields brought by resource bricolage. Hence, this study compensates for the limitations of the existing research and establishes that bricolage can also promote the practices of low-cost breakthrough innovations from new ideas.
(3) The boundary conditions of the impact mechanism of resource bricolage and low-cost breakthrough innovations are discussed, elucidating that the intensity of the indirect impact of resource bricolage promoting low-cost breakthrough innovations through exaptation is regulated by organizational agility. In the context of emerging markets, Chinese manufacturing enterprises attain resource optimization matching and new function discoveries *via* resource bricolage. Specifically, only under high-level organizational agility (marketing and operational agility) can exaptation be better promoted to drive breakthrough innovations. Previous studies on the path adjustment mechanism of breakthrough innovations focused on environmental dynamics (Xi et al., 2017), market turbulence, and other external factors (Mastrogiorgio and Gilsing, 2016). This study incorporates organizational agility into the theoretical model of resource bricolage–exaptation–low-cost breakthrough innovations. The findings revealed that low-cost breakthrough innovations are produced under the comprehensive influence of resource bricolage, organizational agility, and exaptation, which not only conforms to the opinion that resource-based theory about the external environment, internal capabilities, and resource matching plays a role in the practices of strategic change but also reflects the ways of manufacturing enterprises of emerging markets to piece and store heterogeneous and complementary knowledge to seek new external opportunities, realizing the development concept of breakthrough innovations through the complex collaboration of existing products, environments, and users (Andriani et al., 2017).

### Practical Implications

This study has crucial implications for the breakthrough innovations of Chinese emerging markets enterprises. First, it is important for enterprises to focus on the resource bricolage strategy and promote low-cost breakthrough innovations. Moreover, it is significant to apply the bricolage theory to new startups, young business scenarios, large- and medium-sized manufacturing enterprises, informal enterprises, and other mature enterprises to implement bricolage strategies (Yu et al., 2017). Emerging markets manufacturing enterprises cannot wholly rely on the bricolage strategies to spearhead organizational innovations; instead, they should combine the favorable conditions and unfavorable factors of the supply and demand of Chinese emerging markets, and effectively use strategic thinking and operating strategies of resource bricolage to address the issues concerning resource constraints of manufacturing enterprises, weak consumption power, and the pursuit of high-quality and low-cost products and services, thereby helping to promote enterprises to break the fixed mindset, focus on experimentation, exploration and change, and drive breakthrough innovations. Second, implementing an exaptation strategy should be stressed to provide new ideas for low-cost breakthrough innovations. As exaptation can deliver the positive impact of resource bricolage on low-cost breakthrough innovations, enterprises must focus on the critical value of exaptation and create an expansion pool from user interaction, industry–university–research interaction, intellectual capital cultivation, innovation culture construction, and innovation capability enhancement. Meanwhile, enterprises should actively participate in expansion activities and hold expansion forums (Garud et al., 2018) to promote the emergence of exaptation and provide dynamic support for low-cost breakthrough innovations. Third, the key value of organizational agility should be accentuated to provide a capability guarantee for the realization of low-cost breakthrough innovations. Of note, enterprises must quickly perceive changes in the macro-environment and industry development trends through marketing agility and seize new development opportunities to provide environmental support for resource bricolage to promote exaptation and, consequently, promote breakthrough innovations. Alternatively, they must deploy enterprise resources through operational agility, as well as augment resource optimization management, to provide internal driving conditions for the function transfer of products or technologies, and improve the positive impact of resource bricolage on exaptation and low-cost breakthrough innovations. Through the measures mentioned above, decision-making and ideas for low-cost breakthrough innovations can be provided for manufacturing enterprises in emerging markets.

### Limitations and Outlook

This study has some limitations. First, this study obtained sample cross-sectional data through questionnaire surveys, which must be further tested in terms of the longitudinal causality among the characterizing variables. In addition, the questionnaire design plan should be improved in future research to conduct longitudinal empirical research on multiple time nodes. Second, this study did not distinguish the impact of different bricolage types on breakthrough innovations, as different bricolage classifications have different specific forms and applicable fields. Hence, the impact mechanism of bricolage on breakthrough innovations could be investigated separately based on different types of bricolage forms. Third, when discussing the mediating relationship between bricolage and breakthrough innovations, based on reverse innovation thinking, an exaptation mediating variable was used to reveal the mediating mechanism of the correlation between bricolage and breakthrough innovations. Other variables, such as absorptive capacity, compound basic view, can also be investigated in terms of their transmission mechanism of the correlation between the two, where the research on bricolage and breakthrough innovations mediating mechanism can be expanded and enriched further. Finally, restriction of research to statistical methods only. The conducted statistical analyses confirmed a correlation between the variables. However, the study does not indicate to what extent the individual variables affect the entire dependency mechanism and what factors constituting the individual variables are vital from the standpoint of achieving low-cost breakthrough innovations. Presumably, this is an issue that will be discussed in future publications. Simultaneously, owing to the limitations of quantitative inference, future studies can use a combination of qualitative and quantitative methods to make up for these limitations.

## Data Availability Statement

The original contributions presented in the study are included in the article/supplementary material, further inquiries can be directed to the corresponding author/s.

## Author Contributions

CT was mainly responsible for the collation of the manuscript data and the testing of research hypotheses. All authors contributed to the article and approved the submitted version.

## Conflict of Interest

The authors declare that the research was conducted in the absence of any commercial or financial relationships that could be construed as a potential conflict of interest.

## Publisher’s Note

All claims expressed in this article are solely those of the authors and do not necessarily represent those of their affiliated organizations, or those of the publisher, the editors and the reviewers. Any product that may be evaluated in this article, or claim that may be made by its manufacturer, is not guaranteed or endorsed by the publisher.
